# Capturing Reflections for Personal and Professional Development in Medical Education: A Mixed Methods Study

**DOI:** 10.12688/mep.20957.2

**Published:** 2026-03-19

**Authors:** Deanne Spek, Marieke J.J. Ermers, Megan M. Milota

**Affiliations:** 1 Faculty of Industrial Design Engineering, Delft University of Technology, Delft, The Netherlands; 2 Department of Psychiatry, University Medical Centre Utrecht, Utrecht, The Netherlands; 3 Department of the Julius Centre, University Medical Centre Utrecht, Utrecht, The Netherlands

**Keywords:** personal and professional development, reflection, mixed methods, retrospection, intervention

## Abstract

**Background:**

To prepare medical students for their future role in addressing complex health problems, medical education should pay attention to students’ Personal and Professional Development (PPD). Meaningful reflection plays an essential role in such education. We aimed to explore how to facilitate PPD-related reflection, collation and periodic retrospection by medical students.

**Methods:**

We performed an intervention study with fourth-year medical students at the University Medical Centre Utrecht in 2024. The interventions consisted of workshops and individual assignments addressing PPD with three different formats for reflection (note to self, core value or representative item), collection (online or analogue), and retrospection (compilation, value mapping or self-scoring). These were analyzed using a convergent mixed methods design with data from Likert scales and open questions in a survey, focus groups, and analysis of the submitted reflection materials.

**Results:**

Thirty-four students completed the intervention (participation rate 100%), 33 students completed the survey (response rate 97%). The format of making a note to oneself using text/video/audio/image was experienced as the most suitable form of reflection. An online medium was preferred for the collection of reflections, but ease-of-use and an overview display option of the collected materials were deemed crucial requirements. Students experienced the retrospection systems as useful, fun, and actionable and most appreciated the opportunity to trace their personal development.

**Conclusions:**

Students found the reflection, collation, and retrospection methods useful and desirable. Most important for the future design of education is the freedom to choose and adapt, as well as a balance in time investment and perceived added value. Further research should focus on development of a suitable online medium and test this in a longitudinal setting to address retrospection suitability.

## Introduction

Medical education aims to prepare students for a rapidly changing profession that faces many challenges, such as shortages in personnel, scarcity of resources, environmental concerns, and health inequity. ^1, 2^ Simultaneously, future medical professionals are expected to care for their patients and caregivers, themselves, their colleagues, and the environment. Helping students become professionals who are prepared to address these challenges and responsibilities requires more than a transfer of medical skills and knowledge; it demands attention to students’ Personal and Professional Development (PPD) as well. ^3– 6^

Reflection plays a central role in PPD, both in becoming and being an adaptive lifelong-learning healthcare provider. ^3, 5, 7– 10^ *Meaningful* reflection can help students move past superficial learning to deeper, or transformative learning experiences, ^11^ and can arguably only occur when there is enough freedom in both the topic and the format of such reflections. ^4, 8, 12, 13^ Mandatory reflective products or a restricted definition of ‘good’ reflection risk inadvertently training students to behave like ‘reflection zombies’, writing what they think is expected from them rather than recording their authentic impressions and thoughts. ^12, 13^ This does not mean reflective assignments should be abandoned altogether, though. By documenting something about their learning process, students may be able to enrich or deepen earlier reflections, ^14^ track their developing thought processes and progress ^12^ and even turn these reflections into actionable intentions and behaviors. ^11^ We contend it is valuable and necessary to capture and preserve ‘something’ in PPD education precisely because it is an ongoing process. For students to be aware of their PPD, they also need dedicated time and space to collect their reflections at multiple moments in the educational trajectory. ^10, 12^ Education developers have reported on options such as portfolios, artistic capstones, and written reports for PPD. ^3, 10, 15, 16^ Driessen ^17^ nevertheless contends that medical education has a long way to go to implementing educational practices which support valuable reflection. Lingering uncertainties remain regarding how to facilitate the required freedom for PPD reflections, as well as the most suitable format for collecting and periodically returning to these reflections. ^12, 13, 15, 18, 19^ To explore how best to facilitate PPD-related reflections, collation, and periodic retrospection by medical students, we performed a mixed methods intervention study. Our aim can be addressed in three sub-questions:

• RQ1: Which format is most suitable to capture reflections: notes to self (text, audio, video, visual), core values (text and/or visual), or representative items (image, word, object, emotion, etc.)?
• RQ2: Which collection medium is most suitable for collating and organizing reflections (online or analogue)?
• RQ3: Which retrospective system for tracing one’s PPD development is most suitable (story compilations, value mapping, or self-scoring)?

A mixed methods study allows for capturing differences between the different forms of reflection, collection, and retrospection while also understanding why these differences exist from the students’ perspectives. Since reflection is inherently subjective, this study emphasizes the qualitative experiences of students.

## Method

**Figure 1.**
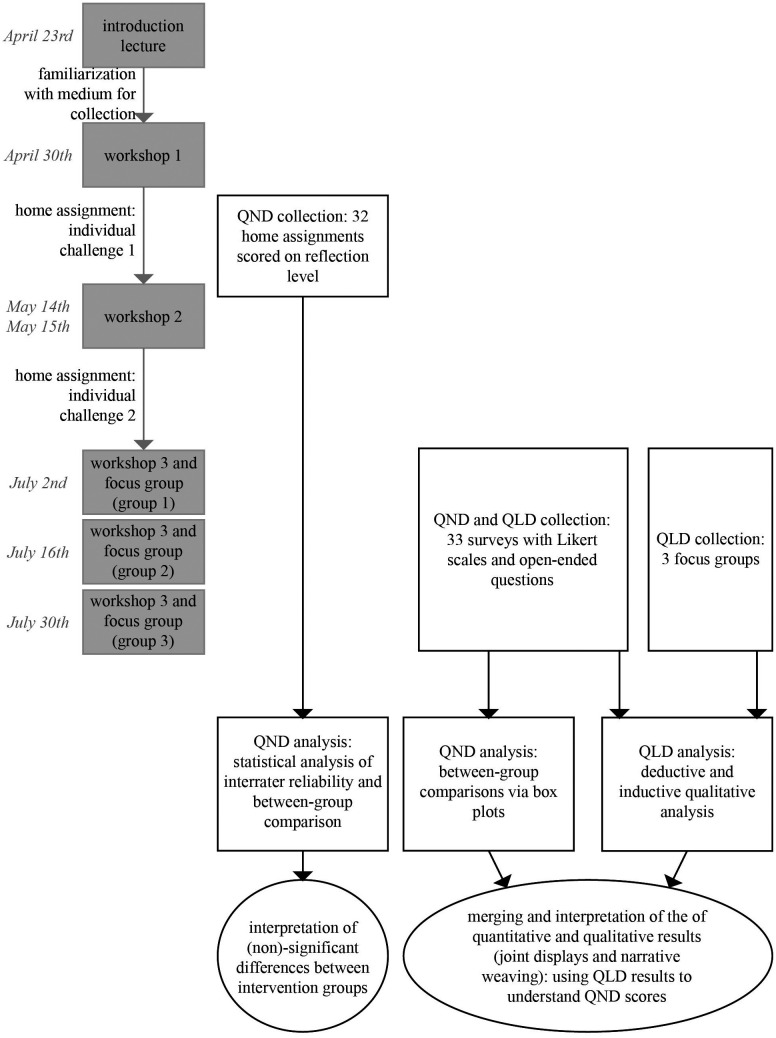
Mixed methods convergent design, parallel data collection (QND = quantitative data, QLD = qualitative data).

The COREQ guidelines ^20^ were used for reporting this study. ^21^ We employed a convergent mixed methods intervention study, ^22^ which involved the parallel collection and analysis of qualitative data (QLD) and quantitative data (QND). ^23^ Students’ reflections were analyzed to assess comparability of reflection levels between intervention groups for combined analysis ( Figure 1).

**Figure 2.**
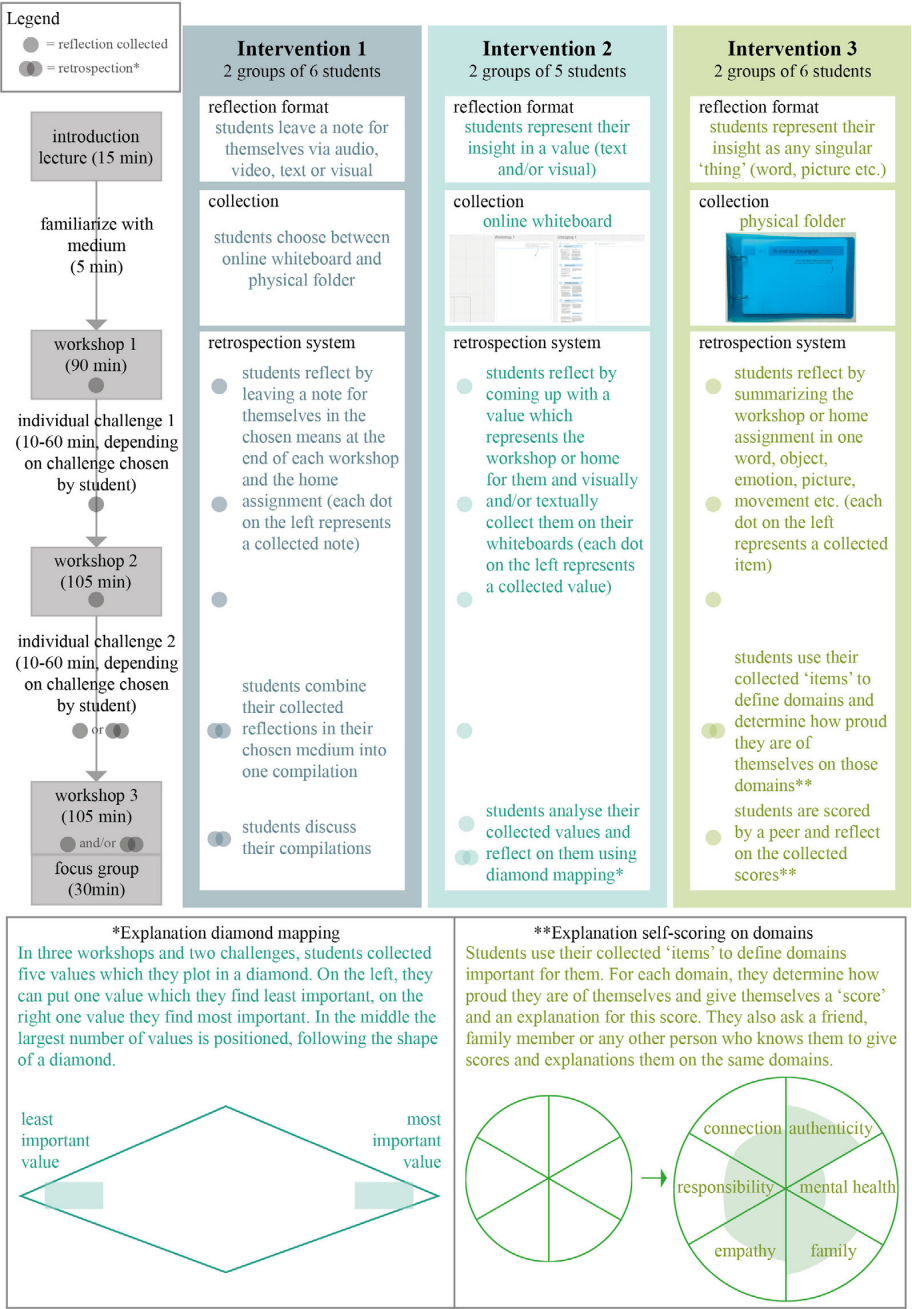
Intervention design and set-up.

The intervention set-ups were developed in a series of co-design sessions ^24^ with students and recent graduates, participants created several prototypes for the reflection format, collection medium, and retrospection system. The first author used insights from literature, discussions with staff and previous research ^18^ to transform these designs into the three interventions, shown in Figure 2. The co-design sessions and literature analysis also informed a set of design principles ( Table 1) which were used to develop the survey and guide the first step of the data analysis.

**Table 1.** Design principles for supporting PPD-related reflections, collation, and periodic retrospection.

Topic	Design principles	Explanation
*Principles for the reflection format*		
Added value	The reflection format must support the students and not be a goal in itself.	Documenting insights to just document is one of the biggest possible downsides of collecting materials. The reflection format must be a supportive means for students, not a goal to deliver.
Suitability	The reflection format must allow students to express and collect what they want to express/collect.	The take-away must not project any ideal form or content to be collected. Students must feel free to ‘document’ what they deem important.
*Principles for the collection medium*		
Personalization	Students have to be able to adapt the collection medium to make it their own.	Colors, shapes, fonts or other aspects of the collection place have to be adaptable to the demands of the student. The collection place has to feel as if it is from the student, not from the university.
Overview	Students have to be able to get a clear overview of the materials they have collected on/in the medium.	The collection place must make it easy for the student to oversee the materials gathered, without too much clicking, flipping through pages or other means of navigating.
Ease-of-use	Students have to be able to easily access, use and manipulate the collection medium.	Insights about oneself can arise during the workshop, during clerkship or at any other moment. Students must be able to access the tool at that moment, or easily and quickly add notes or pictures at a later moment.
*Principles for the retrospection system*		
Individuality	The retrospection system must make it possible for students to trace or see their individual journey without limitations or prescriptive visions of how it should be.	Many journeys and choices are possible for students to become the medical professional they want to become. Instead of good-wrong or a predefined ideal to be reached, the retrospection system must express openness and complexity.
Comparing with oneself	The retrospection system must enable the student to see their own development over time (compare present self to the past self and not to another student).	To monitor growth and development comparison between what the student desired and what has happened must be facilitated. Preferably, the retrospection system is reduces students’ need to compare with others.
Actionable intentions	The retrospection system must support students in formulating actionable lessons or insights.	The moment of retrospection is also the moment to define steps or goals for the next period. The retrospection system must support students in extracting actionable insights from the collected materials.
Meta level reflection	The retrospection system must allow students to take a broader helicopter view about their own development and formulate higher level insights.	Instead of summarizing or enumerating collected insights, the retrospection system must allow for integration of the insights and development of new insights on a higher level. The retrospection system must allow students to take a so-called helicopter view.

Both online and physical collection formats were provided. Miro—a free digital whiteboard platform—was used for the online medium. Students were given access to a personal Miro page including a 2-minute introductory video and existing templates that could be used during the workshops and individual challenges. The analogue medium consisted of an A5 physical folder filled with worksheet materials.

### Setting and participants

We received permission to conduct a pilot with fourth-year medical students at the University Medical Centre Utrecht (N = 34) doing their clerkships in psychiatry, neurology and geriatrics. The pilot education was given as part of the regular education, and participant selection therefore didn’t play a role. The interventions were provided as substitute for small-group sessions with a professional coach in six subgroups. As part of the intervention, students participated in three workshops with their subgroups (topics: norms and values, energy balance, and boundary setting) and chose one of the 4 individual challenges and corresponding reflections to complete at home (see Figure 2). A sample workshop and corresponding challenge options can be found in the Extended Data.

Workshop facilitators consisted of fifth- and sixth-year students and recent graduates. They were recruited by the first author via an information session or email. Those who agreed to act as facilitators received a video and manual explaining the intervention set-up for the group(s) they were facilitating and could contact the first author in case of questions. For both students and facilitators no differentiation in gender was made, as the small sample did not allow for anonymous data collection when asking for gender.

### Reflection analysis

Students’ submitted reflections from the individual challenges were analyzed using an adapted version of the Reflection Evaluation For Learners’ Enhanced Competencies Tool (REFLECT). ^25^ REFLECT is originally created to evaluate written reflections. Since the students’ reflection in this study could be in written, audio or visual formats, we removed the categories of the rubric that were specific to written formats (see Extended Data). After two rounds of independently scoring ten challenges and meeting to compare results and discuss differences, all submitted challenges were independently scored with the final REFLECT version by all authors.

REFLECT scores were analyzed using SPSS 29.0.2.0 for Windows (IBM Corp., Armonk, N.Y., USA). The Intraclass correlation coefficient (ICC) estimates and their 95% confident intervals were calculated based on a mean-rating (k = 3), absolute-agreement, 2-way mixed-effects model. ^26^ There was a good agreement between the authors ^26^ with an ICC of 0.88 (95%CI 0.77–0.94). Differences in scores were resolved by taking the mean. Homogeneity of variance between the groups was established and data were normally distributed, therefore significant differences between groups were calculated using a one-way ANOVA.

### Data collection

A survey was created for this study (Extended Data), combining 7-point Likert scales (QND) with open questions about the given score (QLD). Students completed the survey in the last 10 minutes of the final workshop.

Focus groups were held to deepen our understanding beyond the predefined questions. The first author guided the focus groups, using a semi-structured interview guide (Extended Data). Workshop facilitators were not present at the focus groups, and participants only knew the first author from the introduction class and informed consent procedure. Focus groups lasted 30 minutes, were audio recorded and transcribed verbatim.

### Data analysis

QLD was analyzed Atlas.ti 24.1.1 for Windows using reflexive thematic analysis, ^27^ which included both deductive and inductive coding rounds. ^28^ After familiarization with the data, the design principles ( Table 1) were used to code deductively. All authors analyzed one focus group and 33% of the surveys using the first code tree. Codes were added inductively if student opinions did not fit the predetermined codes. The authors then discussed their inductive codes and collated them in a second code tree. These codes were used by the first author to (re)code all data, after which uncertainties were discussed by all authors. Subcodes were created by the first author for some categories to reach a more nuanced understanding of the QLD ^29^; these subcodes were evaluated by the other two authors and finetuned in discussion.

Differences in Likert scale scores between the groups were visualized using box plots, since the small sample (34 students) limits the possibility to find significant statistical differences (see Extended Data). QLD and QND results were integrated following the approach described by Fetters, Curry, Creswell, ^23^ using both joint displays and narrative weaving of the different results in the text (see Figure 1).

### Reflexivity

The first author is a social design researcher trained in participatory design and research techniques. The second author is a child- and adolescent psychiatrist with experience in qualitative and quantitative research techniques. The last author is a narratologist with expertise in empirical data collection and analysis. The second and third authors are teachers in medical education as well. The interdisciplinary nature of the research team resulted in a diversity in perspectives and interpretations of the datasets. To ensure reflexivity, the research team regularly discussed their assumptions and analytic decisions, kept reflexive logbooks during the coding and analysis process, and compared independent coding rounds to minimize individual bias. As educators and researchers we advocate for more PPD in our medical program; in this study we tried to foreground participant opinions regarding PPD over our own, but we acknowledge our investment in this topic.

## Results

**Figure 3.**
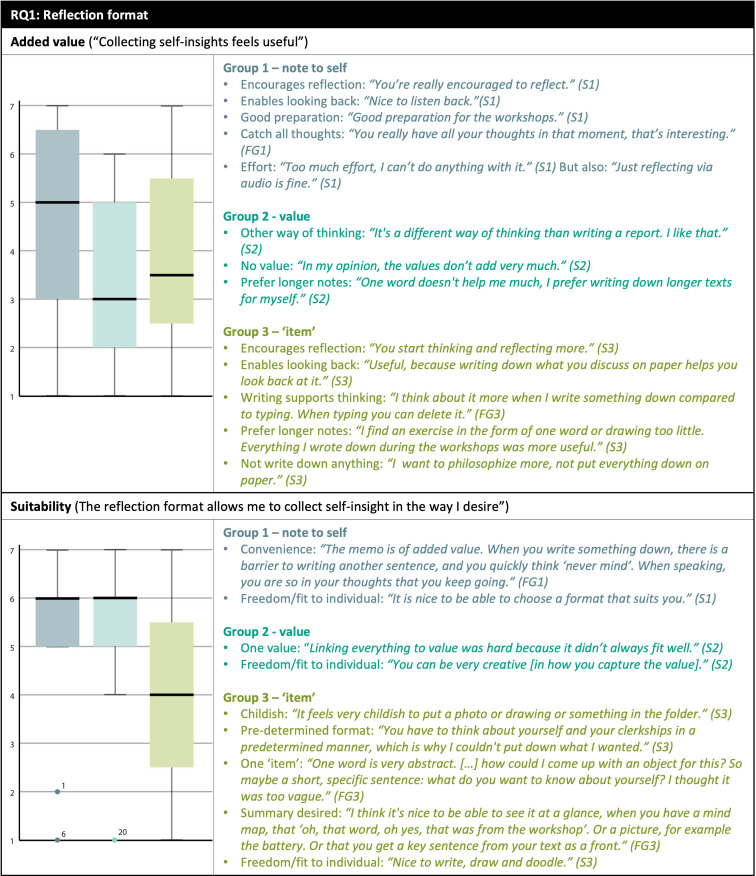

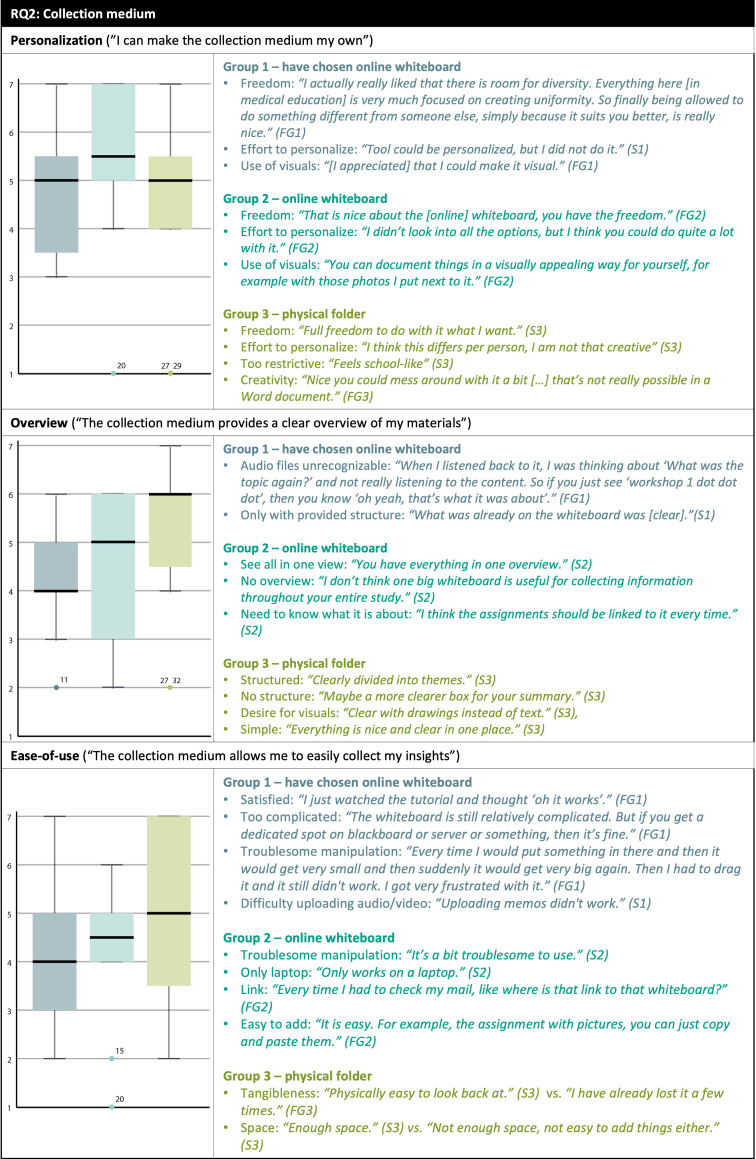

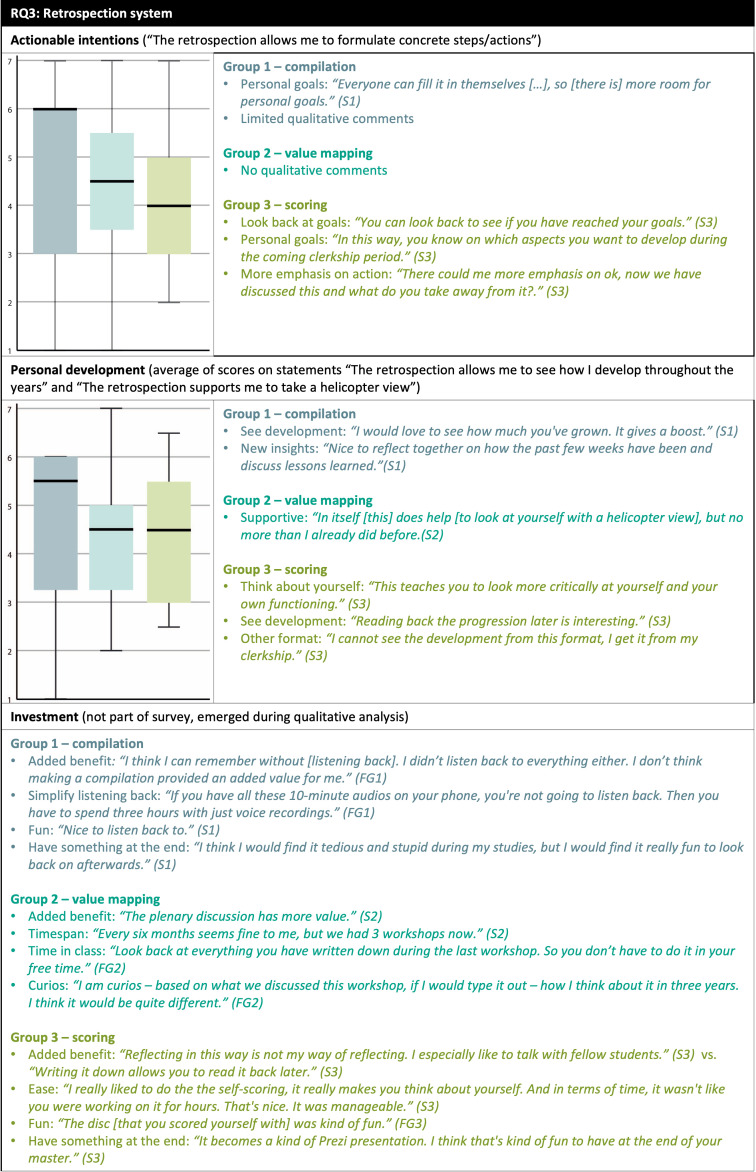
Student opinions on the reflection format, collection medium and retrospection system. Likert scale statements are bracketed in the subtitles, students were asked to give a score ranging from 1 (fully disagree) to 7 (fully agree). Colors differentiate between groups, FG = focus group, S = survey.

All 34 students agreed to participate in the research and signed the informed consent, resulting in 12 participants in group 1 (seven joined the focus group), 10 in group 2 (seven joined the focus group), and 12 in group 3 (nine joined the focus group). 33 students completed the survey (response rate 97%). Figure 3 illustrates the most important insights in students’ opinions on the reflection format, collection medium, and retrospection system using joint displays. Each joint display addresses a code by showing the box plot and subcodes found in each group, further elaborated on below. A more elaborate explanation of the results for education developers is provided in Extended Data.

### Reflection analysis

32 of the 34 students voluntarily submitted their individual challenges for the reflection-analysis (response rate 94%). The reflections demonstrate a wide variety of mediums used within each reflection format. A one-way ANOVA of the REFLECT scores showed no significant difference in reflection level between the three groups (F(2,29) = 3.162, p = 0.057). Given the limited sample size and a p-value near the 0.050 threshold, we conducted a Tukey post-hoc test as an exploratory follow-up analysis. This revealed that group 1 (mean = 2.76) and group 2 (mean = 1.89) differ significantly in reflection level (p = 0.046). There were no statistically significant differences between the other groups (mean of group 3 was 2.43).

### RQ1: Reflection format

The format of making a note to oneself using text/video/audio/image was clearly experienced as the most valuable form of reflection, see Figure 3. Students mentioned the minimal effort required and the experience of achieving a deeper reflection by talking to oneself in a memo (particularly in audio format); they also praised the usefulness of the self-notes as preparation for the workshops, and reported enjoying the ability to choose a method. Reflecting in the form of a single item was experienced as least suitable. Some students articulated that the single item helped support new ways of thinking; most nevertheless preferred collecting longer texts from which a summary (e.g. a key word or visual) could be extracted. Collecting items in a physical folder was furthermore perceived by many as childish or too restrictive of a format.

Students in each of the subgroups emphasized the importance of freedom in the reflection format and emphasized that the format should fit individual reflection needs. Students reported experiencing a sense of freedom when given the opportunity to choose the reflection format (e.g. students in Intervention 1 could choose between image/text/audio/video, the other students could choose between visual/text) and topic (e.g. which insights and how elaborately the students captured these).

### RQ2: Collection medium

The online medium appeared to be most preferred. Students assigned to Intervention 1 could choose between a physical and online medium, and 11 of the 12 students chose online. In addition, students using a physical folder made suggestions for an online medium in the focus group. Students wanted to be able to adapt and personalize the online platform, add content of various forms, and work visually. We found no difference between the groups regarding the degree of personalization the medium allows, all center around a score of 5 ( Figure 3). This is reflected in the qualitative data, which also demonstrate students perceived personalization to be of importance for a reflection collection medium. Ease-of-use and the ability to gain an overview of the collected materials were also praised. Some of these opinions ran contrary to the preference for freedom in adaptation, such as a desire for a medium with more structured themes or sections. Several students experienced the online platform as too complicated to manipulate. An example specific to the Miro online environment was the difficulty in uploading audio/video files.

### RQ3: Retrospection system

Most students appreciated the retrospective facet of the intervention, as it allowed them to see their personal development, think about themselves, and gain new insights. Multiple students expressed a desire to make retrospection insights actionable; some mentioned that they missed practical steps on how to do so in this study design. The design principle on individuality was left out from Figure 3, as several students did not understand the question. In addition, the items on comparing with oneself and meta-level reflection showed similar response patterns and were therefore combined into a new item, *personal development*, based on the average of both scores. Furthermore, student feedback emphasized the importance of finding balance in investment and perceived payoff. The first retrospection system—making a compilation of collected self-notes—took time to complete as it required listening to or rereading all previous reflections. While most students considered this reasonable as it helped them see how their own past selves were thinking, some thought they would be able to recall their experiences without relistening. The second retrospection system of value mapping was not in balance as it was seen as supportive but not especially beneficial. Students did not consider it a productive means of tracing how their past selves were thinking, nor did it create actionable self-insights. The third retrospection system, which consisted of scoring oneself on self-determined domains, was considered a fun exercise. Students particularly valued the fact that this retrospective system included soliciting and discussing input from others. Students reported that this system also provided new insights about themselves, and helped to make these insights actionable. While the third retrospection system scored lowest on the criterium of ‘actionable intentions’ in the quantitative data in Figure 3, qualitative comments show otherwise. Lower scores may be caused by the fact that this questions was answered by all students in group 3 and only by a limited number of students in the other groups; this could be because this was one of the last questions of the survey.

## Discussion

Helping students become professionals prepared to address a wide range of responsibilities and challenges requires attention to their Personal and Professional Development, ^6, 7, 30^ and reflection plays a key role. ^31, 32^ Due to gaps in the literature on *how* to support such reflection, we set out to explore how best to facilitate PPD-related reflections, collation, and periodic retrospection by medical students. With this study we add new insights from the student perspective on suitable formats for a reflection system, possible design requirements, and suggestions for further experimentation on supporting PPD-reflection in medical education.

Our study confirms earlier assertions that reflection should be meaningful ^10, 11^ and that such meaningful reflection is most likely to occur when there is ample freedom in reflection topic and format. ^4, 8, 12, 13^ The opportunity to choose between audio, video, text and/or image facilitated such freedom. In particular, audio diaries were praised by students for their convenience and ability to help them capture all relevant aspects of a thought-process or experience. This is in line with findings of Neve, Lloyd, Collett ^16^ who concluded that audio-diaries were beneficial in bridging the gap between a student’s lived experiences and professional development. Some students reported reaching deeper reflection via writing, and a few even experimented with drawing. Providing alternatives to written or verbalized reflections may benefit those who reflect most effectively via other forms of expression. ^3, 12^ Regardless of the chosen format, our findings show that it should capture more than one item or word. More research is needed to investigate the nuanced differences between more elaborate reflections in written, verbal or non-verbal formats, as our study population was too small to make such a comparison.

The perceived benefits of seeing one’s personal development ^11^ were most pronounced in students looking or listening back to their self-notes. However, students who reflected using the self-scoring system expressed the fun they had and the possibility it gave them to start defining concrete steps or goals. We suggest studying the suitability of using self-notes for collecting and deepening reflection, ^15^ but providing self-scoring as the retrospection system. Input from the self-notes could then be used to define self-scoring domains. Such research should include a longer period of collection before the retrospective exercise than we used in our study. Future research could allow for mixing and matching different formats to gain more nuanced insights into which specific combinations of reflection format, collection medium and retrospection system are most valued by students.

Finally, the results indicate that allowing students to collect reflections in a variety of forms can best be facilitated in an online medium. Indeed, several existing PPD-courses use online portfolios. ^12, 33, 34^ An online medium allows for the automatization (e.g. in transcribing audio) and structure (e.g. in folders or visuals) that students in our study valued. However, ease-of-use is an important requirement for students to benefit from the online medium. Future research should take these considerations into account in developing a suitable online medium for collection of reflections.

In our study, senior students and recent graduates facilitated the education because our earlier studies showed the beneficial effect of near-peer guidance on openness, honesty and growth of the students. ^35, 36^ It may be valuable for future research to further investigate this effect of this form of facilitation the depth of reflection.

### Strengths and limitations

Combining and comparing the qualitative and quantitative results allowed us to gain a more comprehensive understanding of the suitability of various reflection formats; this study thereby provides more valid guidelines for medical educators. Joint displays enabled us to analyze the qualitative and quantitative data side by side, and this narrative weaving technique helped us develop a comprehensive understanding of the results.

Another strength of this study is the practical applicability of *how* to provide PPD-related reflection. The high participation and response rates and non-biased selection of students are additional strengths of this study. Transferability of our findings is enhanced by the modular structure of the interventions, which allows other educators to pick and adapt the insights as needed.

The small sample size and limited duration are limitations of our study. While the retrospection systems were designed to be used every half a year, they were now applied after a few workshops and individual challenge assignments. The condensed form of this pilot may have influenced students’ perceived workload and usability for their personal growth. For example, the focus groups indicated that students would like the retrospection systems more if they were done after half a year. Further longitudinal research is necessary to validate this and investigate other effects of timing.

Another limitation concerns the evaluation materials used. The survey was based on the input of literature and recent graduates and checked by the two other researchers, but it was not piloted with a student sample. The Extended Data provides more insight in the survey for future research. Furthermore, the REFLECT rubric was originally developed for assessing reflective writing. ^25^ Removing two of the categories for this study limits the validity of the rubric. However, our aim was not to compare our results with existing studies but to compare different forms of reflection provided to the students, and the same rubric was used in all three groups.

### Implications for practice

In line with previous research ^4, 12^ we suggest acknowledging and embracing diversity in students’ reflections. This entails augmenting existing written reflective practices that are beneficial to some students with additional formats such as audio or drawing ^12.^ There is not one approach that will work for every student, and students deserve the opportunity to discover which approach fits them best. Medical educators should support students in exploring various forms of reflection to find an approach that supports their lifelong learning. ^3, 12^

The design principles described in Table 1 and those added by the qualitative analysis could be used as the basis for developing the reflection formats, collection mediums, and retrospection systems best suited to the digital infrastructure and curriculum outline of individual institutions. For such experimentation and implementation, medical educators should account for the adjustment time many students may need to become accustomed to and comfortable with creative modalities and a high degree of freedom of choice. ^3^ However, our study also suggests that there will probably always be students who do not see value in reflection or retrospection. Our conversations with recent graduates and research ^10, 35^ both indicate that value may sometimes be perceived only at the end of one’s medical studies. Therefore, we advise making reflection and collection mandatory, albeit without predefining how it should occur. ^3, 12^

## Ethics and consent

We received ethical approval from the NVMO (Dutch Association for Medical Education) Ethical Review Board for this study (study number 2023.8.3) on January 26th, 2024. The authors assert that all procedures contributing to this work comply with the Helsinki Declaration of 1975, as revised in 2024. Surveys were completed anonymously, and transcripts of the focus groups as well as the challenges submitted for the reflection-analysis were anonymized by the first author. Written informed consent for publication of the participants details was obtained from the participants.

## Data Availability

Qualitative data generated and analyzed during the current study cannot be sufficiently de-identified and therefore cannot be made publicly available due to ethical considerations. The following data are made available:
• REFLECT codes (raw underlying data of REFLECT scores) • QND survey (raw underlying data of Likert scale scores from survey) • Codebook (final codebook of qualitative data analysis) This data can be found at: DANS Data Station Life Sciences (DataverseNL): Capturing Reflections for Personal and Professional Development in Medical Education: A Mixed Methods Study. https://doi.org/10.34894/NDEE3P. ^21^ Codebook data are available under the terms of the Creative Commons Zero “No rights reserved” data waiver (CC0 1.0 Public domain dedication). REFLECT codes and QND survey data are only provided after requesting the data via the following steps:
1. Fill in the Data Request Form of UMC Utrecht. By Filling out the Data Request Form you give UMC Utrecht your consent to process your personal data and the information that is submitted to handle your request. UMC Utrecht shall keep all information confidential and process the data in accordance with the GDPR. 2.
The Data Access Committee of UMC Utrecht will handle the request. Depending on the dataset and the request, the handling of your request can take 1 to 6 weeks. Both the fact that your request has been received and whether or not it is granted, will be communicated to you. To assess your request, the Data Access Committee uses the following criteria:
a. the application for issuance is made by an applicant from within or outside UMC Utrecht, who works at a scientific institute and aims to use the dataset for scientific research. The applicant is also responsible for the use of the dataset provided. b. the person responsible for the relevant dataset has agreed to the request for issuance; c. it is reasonably likely that the research will lead to new scientific insights; d. the new research question is compatible with the purpose of the initial data collection as defined in the informed consent; e. the use of body material and the described degree of traceability to personal data are necessary for carrying out the research; f. the issuance and use are in accordance with the control rights of the persons concerned and are within the scope of the applicable consent, or that no objection has been raised by the persons concerned. An exception to the above is for applications where a specific review committee has given approval for a dataset. The working method and composition of this committee, as well as the agreements on the reuse of data, must be described in the information associated with the archived dataset. This document must be approved by UMC Utrecht. For more information on data sharing and requesting, visit Research Data UMC Utrecht - UMC Utrecht, or see the Terms tab in DataverseNL. If you have any questions about the procedure please contact: datamanagementumcu@umcutrecht.nl. DANS Data Station Life Sciences (DataverseNL): Capturing Reflections for Personal and Professional Development in Medical Education: A Mixed Methods Study. https://doi.org/10.34894/NDEE3P. ^21^ The project contains the following extended data:
• Workshop 1 example (designed materials of workshop 1, group 1 as an example) • REFLECT (the adapted REFLECT rubric used in this study) • Survey (the survey used in this study, version of group 1) • Semi-structured focus group guide • Statistical analysis Likert scales (results of statistical analysis) • Elaboration of results (more extended description of the themes and codes as well as more detailed differences and overlaps between groups 1, 2 and 3) Extended data are available under the terms of the Creative Commons Zero “No rights reserved” data waiver (CC0 1.0 Public domain dedication). DANS Data Station Life Sciences (DataverseNL): COREQ checklist for ‘ *Capturing Reflections for Personal and Professional Development in Medical Education: A Mixed Methods Study*’. https://doi.org/10.34894/NDEE3P. ^21^ The COREQ checklist was used to assess the focus groups, and the checklist can be found along with the extended data:
• COREQ checklist (checklist for reporting guidelines) ^20^ The checklist is available under the terms of the Creative Commons Zero “No rights reserved” data waiver (CC0 1.0 Public domain dedication).

## References

[1] Frenk J Chen L Bhutta ZA : Health professionals for a new century: transforming education to strengthen health systems in an interdependent world. *Lancet.* 2010;376(9756):1923–1958. 10.1016/S0140-6736(10)61854-5 21112623

[2] Majumder MAA Haque M Razzaque MS : Editorial: trends and challenges of medical education in the changing academic and public health environment of the 21st century. *Front. Commun.* 2023;8:8. 10.3389/fcomm.2023.1153764

[3] Fleer J Smit MJ Boer HJ : An evidence-informed pedagogical approach to support Professional Identity Formation in medical students: AMEE Guide No. 171. *Med. Teach.* 2025;47(4):580–588. 10.1080/0142159X.2024.2387809 39110856

[4] Veen M Croix A de la : How to grow a professional Identity: philosophical gardening in the field of medical education. *Perspect. Med. Educ.* 2023;12(1):12–19. 10.5334/pme.367 36908744 PMC9997106

[5] Verwer S Braak M van : Subjectification in health professions education: why we should look beyond the idea of professional identity formation. In: Brown MEL Veen M Finn GM , eds. *Applied Philosophy for Health Professions Education: A Journey Towards Mutual Understanding* Singapore: Springer Nature Singapore;2022;23–37. 10.1007/978-981-19-1512-3_3

[6] Chandran L Iuli RJ Strano-Paul L : Developing “a way of being”: deliberate approaches to professional identity formation in medical education. *Acad. Psychiatry.* 2019;43(5):521–527. 10.1007/s40596-019-01048-4 30993596

[7] Biesta G : Risking ourselves in education: qualification, socialization, and subjectification revisited. *Educ. Theory.* 2020;70(1):89–104. 10.1111/edth.12411

[8] Mann K Gordon J MacLeod A : Reflection and reflective practice in health professions education: a systematic review. *Adv. Health Sci. Educ. Theory Pract.* 2009;14(4):595–621. 10.1007/s10459-007-9090-2 18034364

[9] Pestka B : Using reflective practice to enhance student professionalism. *J. Med. Educat. Curri. Develop.* 2024;11:23821205241250172. 10.1177/23821205241250172 PMC11084998 38736714

[10] Ede AE van Claessen RJM Gils M van : How to coach student professional development during times of challenges and uncertainties. *BMC Med. Educ.* 2023;23(1):600. 10.1186/s12909-023-04588-4 PMC10463913 37608301

[11] Murdoch-Eaton D Sandars J : Reflection: moving from a mandatory ritual to meaningful professional development. *Arch. Dis. Child.* 2014;99(3):279–283. 10.1136/archdischild-2013-303948 23975720

[12] Croix A de la Veen M : The reflective zombie: problematizing the conceptual framework of reflection in medical education. *Perspect Med Educ.* 2018;7(6):394–400. 10.1007/s40037-018-0479-9 30353284 PMC6283773

[13] Veen M Skelton J Croix A de la : Knowledge, skills and beetles: respecting the privacy of private experiences in medical education. *Perspect. Med. Educ.* 2020;9(2):111–116. 10.1007/s40037-020-00565-5 32026318 PMC7138766

[14] Boud D , Cohen R , Walker D : Using experience for learning. Open University Press;1993. Reference Source

[15] MacAskill W Chua WJ Woodall H : Beyond the written reflection: a systematic review and qualitative synthesis of creative approaches to reflective learning amongst medical students. *Perspect. Med. Educ.* 2023;12(1):361–371. 10.5334/pme.914 37720690 PMC10503530

[16] Neve H Lloyd H Collett T : Understanding students’ experiences of professionalism learning: a ‘threshold’ approach. *Teach. High. Educ.* 2017;22(1):92–108. 10.1080/13562517.2016.1221810

[17] Driessen E : Do portfolios have a future? *Adv. Health Sci. Educ. Theory Pract.* 2017;22(1):221–228. 10.1007/s10459-016-9679-4 27025510 PMC5306426

[18] Ermers M Spek D Beelen S van : Cultivating time and space for becoming: medical students’ experiences on novel education to support subjectification and professional identity formation. [Manuscript in preparation]2024.

[19] Branch WT Jr : The road to professionalism: reflective practice and reflective learning. *Patient Educ. Couns.* 2010;80(3):327–332. 10.1016/j.pec.2010.04.022 20570461

[20] Tong A Sainsbury P Craig J : Consolidated criteria for Reporting Qualitative research (COREQ): a 32-item checklist for interviews and focus groups. *Int. J. Qual. Health Care.* 2007;19(6):349–357. 10.1093/intqhc/mzm042 17872937

[21] Spek D , Ermers M , Milota M : Capturing reflections for personal and professional development in medical education: a mixed methods study. In: DataverseNL,2025. 10.12688/mep.20957.2 PMC13019040 41909087

[22] Creswell JW : A concise introduction to mixed methods research. SAGE Publications, Inc.;2015. Reference Source

[23] Fetters MD Curry LA Creswell JW : Achieving integration in mixed methods designs—principles and practices. *Health Serv. Res.* 2013;48(6 pt 2):2134–2156. 10.1111/1475-6773.12117 24279835 PMC4097839

[24] Sanders EBN Stappers PJ : Co-creation and the new landscapes of design. *CoDesign.* 2008;4(1):5–18. 10.1080/15710880701875068

[25] Wald HS Borkan JM Taylor JS : Fostering and evaluating reflective capacity in medical education: developing the REFLECT rubric for assessing reflective writing. *Acad. Med.* 2012;87(1):41–50. 10.1097/ACM.0b013e31823b55fa 22104060

[26] Koo TK Li MY : A guideline of selecting and reporting intraclass correlation coefficients for reliability research. *J. Chiropr. Med.* 2016;15(2):155–163. 10.1016/j.jcm.2016.02.012 27330520 PMC4913118

[27] Braun V Clarke V : Using thematic analysis in psychology. *Qual. Res. Psychol.* 2006;3(2):77–101. 10.1191/1478088706qp063oa

[28] Fereday J Muir-Cochrane E : Demonstrating rigor using thematic analysis: a hybrid approach of inductive and deductive coding and theme development. *Int. J. Qual. Methods.* 2006;5(1):80–92. 10.1177/160940690600500107

[29] Saldaña J : *The coding manual for qualitative researchers.* 2nd ed. London: SAGE Publications;2013.

[30] Cruess RL Cruess SR Boudreau JD : A schematic representation of the professional identity formation and socialization of medical students and residents: a guide for medical educators. *Acad. Med.* 2015;90(6):718–725. 10.1097/ACM.0000000000000700 25785682

[31] Sandars J : The use of reflection in medical education: AMEE Guide No. 44. *Med. Teach.* 2009;31(8):685–695. 10.1080/01421590903050374 19811204

[32] Hoffman LA Shew RL Vu TR : Is reflective ability associated with professionalism lapses during medical school? *Acad. Med.* 2016;91(6):853–857. 10.1097/ACM.0000000000001094 26760059

[33] Monrouxe LV : Identity, identification and medical education: why should we care? *Med. Educ.* 2010;44(1):40–49. 10.1111/j.1365-2923.2009.03440.x 20078755

[34] Biesta G : Good education in an age of measurement: ethics, politics, democracy. New York: Routledge;2010. Reference Source

[35] Kalet AL Sanger J Chase J : Promoting professionalism through an online professional development portfolio: successes, joys, and frustrations. *Acad. Med.* 2007;82(11):1065–1072. 10.1097/ACM.0b013e31815762af 17971693

[36] Ermers MJJ Spek D Dijk E van : Designing for subjectification in medical education: development and evaluation of the Doctor in Development program.[Manuscript submitted for publication]2025.

