# Supplementary material for: Capturing Reflections for Personal and Professional Development in Medical Education: A Mixed Methods Study
Source: MedEdPublish (2016). 2026 Mar 19;15:227. Originally published 2025 Oct 27. [Version 2] doi: 10.12688/mep.20957.2 (PMC13019040; doi:10.12688/mep.20957.2)
Supplement: Peer review history

# Reviewer report: Reviewer response for version 2

**DOI:** 10.21956/mep.23056.r45620

**Published:** 2026-03-30

**Authors:** Samar Aboulsoud

**Affiliations:** 1 Cairo University, Cairo, Egypt

Thank you for submitting the revised version of your manuscript. I appreciated the opportunity to review this work and commend the authors for addressing the previous round of comments in a thoughtful and systematic manner.

 This manuscript tackles an important and timely topic, namely the role of **meaningful reflection in professional practice development (PPD)**. The emphasis on moving beyond superficial or performative reflection toward more authentic and transformative learning is particularly valuable and well aligned with current discussions in medical education.

**Strengths**

 The revisions have significantly strengthened the manuscript. In particular:

- The **clarification of the research questions** and the inclusion of a rationale for the mixed-methods design enhance the coherence and methodological rigor of the study.
- The **expanded description of the workshops, clerkship context, and thematic content** improves transparency and supports transferability.
- The **refinement of both quantitative and qualitative results**, including clearer differentiation between intervention groups and the inclusion of “actionable” intentions, adds depth and nuance to the findings.
- The **enhanced methodological detail**, especially regarding the adaptation of the REFLECT rubric, subcoding processes, and reflexivity, is commendable.
- The **figures (particularly Figures 2 and 3)** are clear, well-designed, and significantly aid reader comprehension.

Overall, the manuscript demonstrates careful revision and responsiveness to feedback.

**Areas for Further Improvement**

 While the manuscript is much improved, a few areas could benefit from further refinement:

1. **Brevity and Focus**
  Despite efforts to streamline the text, the manuscript remains somewhat lengthy. Further condensation—particularly in descriptive sections—would enhance readability and ensure that key messages are communicated more effectively.
2. **Clarity of Key Concepts**
  The concept of “meaningful reflection” is central to the paper and well introduced in the abstract. However, it may benefit from slightly sharper operationalization earlier in the manuscript to ensure consistent interpretation by readers.
3. **Integration of Literature**
  The manuscript could be further strengthened by engaging with recent literature on reflective practice in medical education. For example:
  - refer to 1

Incorporating such work may help contextualize the findings and reinforce the relevance of the study.

**Conclusion and Recommendation**

 This is a well-conducted and thoughtfully revised study addressing a highly relevant topic in medical education. With minor revisions to improve conciseness and further strengthen conceptual clarity and practical implications, the manuscript will make a valuable contribution to the field.

Have any limitations of the research been acknowledged?

Yes

Is the work clearly and accurately presented and does it cite the current literature?

Yes

If applicable, is the statistical analysis and its interpretation appropriate?

Yes

Are all the source data underlying the results available to ensure full reproducibility?

Partly

Is the study design appropriate and is the work technically sound?

Yes

Are the conclusions drawn adequately supported by the results?

Yes

Are sufficient details of methods and analysis provided to allow replication by others?

Yes

Reviewer Expertise:

CPD, Accreditation, educational leadership, IPE, Medical education Continuum

I confirm that I have read this submission and believe that I have an appropriate level of expertise to confirm that it is of an acceptable scientific standard.

## References

[1] Srijampana VVGR, Darsi K, Potti R, Gujjarlapudi C. Perceptions and practices of teaching faculty on effective usage of reflection in medical education. Journal of Dr. NTR University of Health Sciences. 2025;14(3):323–329.

# Reviewer report: Reviewer response for version 2

**DOI:** 10.21956/mep.23056.r45593

**Published:** 2026-03-25

**Authors:** Prof. Irma Manjavidze M.D.,PhD., Associate Professor. Dali Chitaishvili M.D., PhD.

**Affiliations:** 1 Head of Clinical Skills and Multidisciplinary Simulation Department, Tbilisi State Medical University, Tbilisi, Georgia; 2 Head of Department of Clinical Skills and Multidisciplinary Simulation, Tbilisi State Medical University, Tbilisi, Georgia

We are satisfied with the revisions made by the authors, and we are happy for our decision to be recorded as “Approved.”

Have any limitations of the research been acknowledged?

Partly

Is the work clearly and accurately presented and does it cite the current literature?

Partly

If applicable, is the statistical analysis and its interpretation appropriate?

Partly

Are all the source data underlying the results available to ensure full reproducibility?

Partly

Is the study design appropriate and is the work technically sound?

Yes

Are the conclusions drawn adequately supported by the results?

Yes

Are sufficient details of methods and analysis provided to allow replication by others?

Partly

Reviewer Expertise:

NA

We confirm that we have read this submission and believe that we have an appropriate level of expertise to confirm that it is of an acceptable scientific standard.

# Reviewer report: Reviewer response for version 1

**DOI:** 10.21956/mep.22445.r44377

**Published:** 2025-12-29

**Authors:** Saima Batool

**Affiliations:** 1 University of Lahore, Punjab, Pakistan

The article is well written with detailed evidence from published references. Its strengths are a co-design component and a mixed-method design using the convergent method.

 The authors must look into the following observations: 

 1. describe why the convergent design was used

 2. how the reflexivity was established

 3. The facilitators were senior students, so how was the authors ensure reflexivity due to facilitator-participant proximity?

 4. how they adapted a written reflection rubric REFLECT to visual, audio and video reflections?

 5. How much time was given to each activity, also mention facilitator workload

 6. How was the survey instrument validated?

 7. How they ensure the transferability of findings as there were only Utrecht university students as the participants?

Have any limitations of the research been acknowledged?

Yes

Is the work clearly and accurately presented and does it cite the current literature?

Yes

If applicable, is the statistical analysis and its interpretation appropriate?

I cannot comment. A qualified statistician is required.

Are all the source data underlying the results available to ensure full reproducibility?

Yes

Is the study design appropriate and is the work technically sound?

Partly

Are the conclusions drawn adequately supported by the results?

Yes

Are sufficient details of methods and analysis provided to allow replication by others?

Yes

Reviewer Expertise:

Medical education, Technology enhanced learning, Pediatrics

I confirm that I have read this submission and believe that I have an appropriate level of expertise to confirm that it is of an acceptable scientific standard, however I have significant reservations, as outlined above.

# Response

**Published:** 2026-02-16

**Authors:** Deanne Spek

**Affiliations:** Psychiatry, Universitair Medisch Centrum Utrecht, Utrecht, Utrecht, The Netherlands

We thank the reviewer for her comments that allow us to improve the article. Below, we outline for each of the comments how we addressed the issue. 1. We added a rationale for the mixed methods convergent design at the end of the introduction to accommodate for the comment of this reviewer and the first reviewers. 2. We elaborated our reflexivity statement with an explanation about how reflexivity was supported during the process of analyses. 3. The comment of the reviewer shows that our explanation does not convey what we meant. Facilitators of the workshops were not present during the focus groups with students, to provide students with the freedom to express their voices. The education was evaluated with facilitators separately; the facilitator evaluations were not analyzed in this article. We added the point that facilitators were not present in the data collection section. 4. We provided more explanation about why and how the RUBRIC was modified in the methods section. Building on this adaptation, we elaborated on the limitation of adapting the RUBRIC in the discussion section. 5. We added the workload for students to Figure 2. However, we decided to not add the facilitator workload since this article does not focus on the facilitator role. We believe adding more information would complicate the figure and hinder readability. 6. The survey was not validated but based upon the design principles, as is common in design research. This is explained in the article, but we made sure to clearly mention “a survey was created for this study” in the data collection section to prevent readers from assuming a validated questionnaire was used. 7. We understand the comment of the reviewer on transferability. However, we would like to point out that the difficulty in addressing personal development, subjectification and reflection is widespread according to literature (see introduction). Since this pilot study is a first exploration of possibilities, the student population was limited. Furthermore, we had no previous educational design to build upon. We have addressed transferability more elaborately in the discussion.

# Reviewer report: Reviewer response for version 1

**DOI:** 10.21956/mep.22445.r44378

**Published:** 2025-12-29

**Authors:** David A. Lindholm

**Affiliations:** 1 Uniformed Services University of the Health Sciences, Bethesda, USA

Establishing the importance of reflection as part of personal and professional development in medical students, the authors describe an interventional pilot study of a single-institution, single-clerkship rotation of 4 ^th^-year students involving workshops and individual assignments to explore *how* best to facilitate reflection and periodic retrospection.  Students were assigned to one of 3 formats for reflection (note to self, core value, or representative items), one of 3 formats for retrospection (compilation, value mapping, and self-scoring), and collection medium (online or analogue).  The authors assessed their findings by converging on quantitative and qualitative analyses.  The authors report that their intervention was well-received, and they emphasize the importance of student choice in reflection and by extension, the importance for education programs to be flexible in their approach to reflective practice to optimize its value for individual students.  They also identify areas for future research.  The authors have a clear statement of aims, rigorous methodology, and provide valuable insight on an important topic in medical education—by focusing on the *how*, this innovation provides a practical approach for medical educators.  That said, the manuscript would benefit from increased specificity in describing the intervention and its context, clarifying and expanding their data presentation (e.g., some key areas of focus in the manuscript results/discussion are not included in the key data figure), and ensuring that discussion points/conclusions are not overstated based on the data presented.  The manuscript also contains multiple spelling and grammar errors that need to be corrected.  I have included comments regarding each area of the paper below.

**ABSTRACT**

 Results: “The format of **a** making a note”—remove the extra “a”

**INTRODUCTION**

 -1 ^st^ paragraph: “care for their patients and carers”—is “carers” meant to be careers or caregivers?

 -2 ^nd^ paragraph: “adaptive life-learning healthcare provider”—“life-learning” reads awkwardly…perhaps “lifelong-learning”?

 -2 ^nd^ paragraph: add comma—“…deeper, or transformative learning experiences, and can…”

 -2 ^nd^ paragraph: remove apostrophe in “portfolio’s”—should be “portfolios”

**METHODS**

 -figure 1: “merging and interpretation of the merging of quantitative and qualitative results…”—remove the second “merging of”; otherwise, phrasing is awkward

 -figure 2: the legend explains the meaning of a single circle and two overlapping circles; however, the figure also employs three overlapping circles, four overlapping circles, and circles of different sizes—what’s the significance of these?  There is a lot to take in on the figure, and the figure legend is not clearly shown.

 -figure 2//diamond mapping: “which they 1plot”—errant “1.”  Description indicates that “least important” is on left and “most important” is on right, but the actual diamond shows the opposite

 -table 1//suitability: “what they wat to express…”—should be “want”

 -table 1: Order in the rest of the manuscript is reflection format, retrospection, then collection, but in table 1 (and the figures), it’s reflection format, then collection, then retrospection.  Recommend reconciling/unifying this to decrease cognitive load.

 -table 1//retrospection: “Many journeys and choices are possible to become the medical professional a student wants to become”—consider “Many journeys and choices are possible *for the student to *become…”

 -table 1//retrospection: “to see if you become who you want to become”—this is the only use of the 2 ^nd^ person in the manuscript/table—change to third person for consistency.

 -setting and participants: “students participated in three workshops…and chose an individual challenge to complete at home”—it’s not clear what topics the workshops covered or what was involved in the individual challenge from the manuscript text or figure 2.  The dataverse.nl site for Extended Data is not available for reference.  There doesn’t need to be a great degree of detail on what the topics or challenges were in the text itself, but general ideas would be helpful (e.g., faculty-selected or student-selected challenging cases, ethics, controversial topics, etc).  It would also be helpful to know what clerkship this was implemented on as part of the context, as the relative proportion of negative vs positive comments and even perceived usefulness may vary based on the demands of the clinical rotation.  Figure 2 also shows a set number of students in each of the six subgroups (Group 1a, Group 1b, etc), but it is not clear what they were allowed to choose.

 -“Those who agreed to act as facilitator”—should be “facilitators”

 -“Furthermore, we do not consider gender to be pertinent to our research question”—please include citation from the literature as to why this assumption was made.  There is comment in the discussion about the impact of this aspect as well, so this seems to be self-contradictory.

 “Students’ submitted reflections form the…”—should be “from”

 -Data analysis: “All authors analyzed one focus group and 30% of the surveys…”—shouldn’t this be 33% to get to 100%?

 -Reflexivity”: “…researcher trained participatory design…”—change to “trained in”?  Also, “The second and thirs author”—change to “third”?

**RESULTS**

 Figure 3:

1. Throughout the text, RQ1 is the format for reflection, RQ2 is the retrospective system, and RQ3 is the collection medium.  However, figure 3 shows RQ2 as the collection medium and RQ3 as the retrospective system.  As above, Table 1 and Figure 2 also flip this order.  This needs to be reconciled to be consistent across the entirety of the manuscript and table/figures.  I think the flow in figures 2-3 (reflection format then collection system then retrospective system) makes the most sense intuitively, but consistency matters most to decrease cognitive load on the reader.
2. Figure 3 subcodes match table 1 for reflection format (added value, suitability) and partially match table 1 for collection medium (overview, ease-of-use but not personalization).  However, Figure 3 does not match table 1 for the retrospective system (table 1: individuality, comparing with oneself, actionable intentions, meta level reflection vs Figure 3: personal development).  This should be reconciled or explained as to why these do not match.
3. The data in Figure 3 should be able to stand alone from the text and especially from the extended data.  The text indicates that the authors used a 7-point Likert scale, but the anchors for the Likert scale are not clearly described—consider adding either a y-axis label or a footnote explaining the anchors.  Additionally, the question on which the Likert scale was based is not clear (e.g., was this the extent to which students felt the system improved personal development, for example?).  I do not have a clear sense of what was actually measured, so it’s not clear how to interpret the results or whether the interpretations drawn in the text are justified by the quantitative data collected.

-Reflection analysis: “This revealed that group 1 (mean 2.76) and group 2 (mean 1.89)…”  Looking at Figure 3, all of the Likert scale responses shown were well above 2.76 and 1.89—I’m not sure what these numbers actually mean.  Please clarify what these means represent.

 -RQ1: Reflection format: “The format of making a note to oneself…was clearly experienced as the most suitable form of reflection, see Figure 3.”  Figure 3 shows very similar Likert response for both group 1 and 2.  You could state that the reflection formats in groups 1 and 2 were “clearly” the most suitable forms, but the data does not support the statement that only group 1 was “clearly” the most suitable.  Additionally, when combining the qualitative data, the representative quotes don’t overwhelming show that the group 1 method is best.

 -“Reflecting in the form of a single item or value was not experienced as suitable.”  This statement is not supported by the presented data, as group 2’s Likert scale appears to be non-inferior to group 1, and the qualitative comments for both include both positive and negative comments.

 -“Students in each of the subgroups emphasized the importance of freedom in the reflection format…experience of a sense of freedom when given the opportunity to choose the reflection format.”  Within group 1, there appears to be some degree of choice (audio, video, text, or visual), but there does not seem to be as much freedom of choice within the other groups (also different reflection formats), and the methods don’t seem to indicate that the students got to choose which “reflection format” (group 1, 2, or 3) to which they were assigned.  Would recommend increased specificity in terminology when referring to “reflection format”—is this choice of the which of the 3 format groups they could join or some specific choice within one of those assigned groups?

 -RQ2: Retrospection system: “Students expressed a desire to make retrospection insights actionable.”  None of the representative quotes suggest the desire for actionable insights—would suggest adding quotes to support this assertion.  Indeed, Table 1 has multiple sub-domains (individuality, comparing with oneself, actionable intentions, meta-level reflection), but Figure 3 only lists “personal development” as a subdomain within RQ2, which is not reflected in Table 3.  Would recommend reconciling those, and expanding the subdomains commented on in Figure 3 to match the important results reflected in the manuscript.

 -RQ2: “However, students considered this [listening to/rereading] reasonable as it helped them see how their own past selves were thinking.”  The quotes under investment, though, don’t seem to support this: “I don’t think making a compilation provided an added value.”  “You’re not going to listen back.”  “I think I would find it tedious and stupid…”  The sentence in the results seems to gloss over the fact the presented quotations included a balance of negative comments.

 -RQ2: “The second retrospection system…was considered a fun exercise.”  The quote regarding “fun” comes from group 3, not group 2.

 -RQ2: “The third retrospection system of value mapping…”  Based on Figure 2 and 3, this should be the second system and not the third.

 -RQ3: “…11 of the 12 students who could choose”—would add a parenthetical comment that this was group 1; see above for earlier comments on clarifying who was given choice and what the choices were.

**DISCUSSION/CONCLUSION**

 -“We found that students valued the reflection and retrospection activities…”  Looking at Figure 3 (“added value”), the median Likert score for 2 of the 3 groups was no more than about 3.5…only group 1 seemed to really value the format.  (This is an example of where understanding what the Likert scales were measuring would be helpful.)  Comments were variably positive and negative.  What percent or relative proportion of comments were positive vs negative?  It seems that a more accurate statement would be that some students valued it and some did not.  Were there characteristics that predicted which students valued reflection vs which did not?

 -“…they also expressed the intention to use their reflections as a guide for future behavior”—as above, this data isn’t presented in the manuscript/figure, and it would be helpful to include since it seems to be a major takeaway for the study. 

 -“Providing a reflection format, retrospection system and collection medium that support these benefits outbalances the time and energy they take to complete.”  This seems to be dependent on the medium provided.  For example, based on Figure 3/investment, students state that compilation took too much time, discussion was more valuable than value mapping, and the frequency of it was too high.  The info presented does not make it clear that the benefits outbalance the time/energy required.  Indeed, it seems that a big barrier that educators would need to overcome when implementing a curriculum like this  is to figure out how to move students from seeing the activities as “tedious and stupid” to finding value afterwards.

 -“With this study we add new insights…”: it seems there are three areas of insights (suitable formats, possible design requirements, and suggestions for further experimentation).  If the intent is 3 areas, consider adding a comma between “reflection system” and “possible design.”

 -“Our study confirms earlier assertions that reflection should me meaningful”—change to “be meaningful.”

 -“such meaningful reflection can only occur when there is enough freedom”—yet, only 1/3 of the students had choice (group 1); did only 1/3 of the students find it meaningful?

 -“…the possibility it gave them to start defining concrete steps or goals”—again, actionable, specific items is a recurring theme, but the data presented in the figures do not include this.  Suggest adding this to figure 3 since it is such a major emphasis.

 - “We suggest studying the suitability of using self-notes for collecting and deepening reflection, but providing self-scoring as the retrospection system.”   From Figure 3/personal development, it appears that compilation (also part of group 1/self-notes) is significantly higher on the Likert scale than value mapping and scoring; value mapping and scoring seem to have about the same median Likert scale.  Based on the data presented, it’s not clear why group 2’s approach (value mapping) is not favored at the same level as group 3 (scoring).  A limitation that is also highlighted by this sentence is that there was no mixing and matching of reflection format, collection medium, and retrospection system—indeed, would student opinions have been optimized had there been further mixing and matching (e.g., reflection format of group 1, collection medium of group 2, retrospection system of group 3)?  I recognize that mixing/matching was not the design of the study, but it is a limitation worth mentioning, especially given that one conclusion is that freedom and adaptability are critical.

 -“…use online portfolio’s”—portfolios should be plural, not possessive.

 -“Another strength of this study is the practical applicability of how to provide PPD-related reflection are strengths of this study.”  This sentence includes redundant phrasing—recommend streamlining.

 -“Gender and sex may influence students’ preferences…” and “…show the necessity of freedom and flexibility regardless of sex and gender…”  This is emphasized in the discussion, yet the authors previously asserted (without supporting evidence) that “we do not consider gender to be pertinent to our research question.”  Since this is emphasized again in the manuscript, it seems like it is relevant, and it is important to note as a limitation since it was not assessed.  Additionally, one cannot make a conclusion that “freedom and flexibility” are necessary “regardless of sex and gender” if one did not measure those items.

 -The Extended Data may provide more insight in the survey for future research.”  This sounds like an unevaluated claim.  Does it or does it not?  The “may” in this statement makes it seem as though the extended data have not been assessed.

 -“However, our study also confirms that there will probably always be students wo [spelling] do not see value in reflection or retrospection.”  Agree this is likely true; however, students were assigned to one group or another, and their actual freedom to choose which reflection format they used was very limited (they could choose a sub-format within group 1 but could not decide group 1, 2, or 3).  Because there was actually limited choice within the design of the intervention over a finite/limited time period, the authors can’t state that the study confirms this.

Have any limitations of the research been acknowledged?

Yes

Is the work clearly and accurately presented and does it cite the current literature?

Partly

If applicable, is the statistical analysis and its interpretation appropriate?

Partly

Are all the source data underlying the results available to ensure full reproducibility?

Partly

Is the study design appropriate and is the work technically sound?

Yes

Are the conclusions drawn adequately supported by the results?

Partly

Are sufficient details of methods and analysis provided to allow replication by others?

Partly

Reviewer Expertise:

Within medical education, I have focused on faculty development, feedback, and curriculum development.

I confirm that I have read this submission and believe that I have an appropriate level of expertise to confirm that it is of an acceptable scientific standard, however I have significant reservations, as outlined above.

# Response

**Published:** 2026-02-16

**Authors:** Deanne Spek

**Affiliations:** Psychiatry, Universitair Medisch Centrum Utrecht, Utrecht, Utrecht, The Netherlands

We thank the reviewer for his elaborate feedback, which helped us enhance the quality and readability of the article. We address the feedback in a summarized manner below since several comments referred to the same issue.

- We appreciate that the reviewer points out several spelling/grammar mistakes and we have carefully reread the whole article to remove any additional mistakes not yet mentioned by any of the reviewers.
- We agree with the reviewer that for the reader and from the point of view of the students, the order of the research questions should be changed to reflection format, collection medium, and retrospection system. We applied this change in the whole article, including the figures and tables, to address several of the useful comments made by the reviewer.
- METHODS-figures: Besides improving the grammar, we improved readability of Figure 2 using the comments of the reviewer as a starting point. We adapted the legend, simplified the icons (circles) used, and removed the information about subgroups a and b since this information is redundant for the reader.
- METHODS-setting and participants: We are sorry to hear that Dataverse was not available at the time the reviewer tried to access it. As far as we know, the extended data is now fully accessible. We also addressed the reviewer’s suggestion to give a general idea of the topic covered in the workshops and we added information on the type of clerkships the students were doing.
- METHODS/DISCUSSION-gender: We much appreciate the reviewer’s comment about our seemingly contradictory descriptions about the importance of gender. The main reason for not collecting information about gender was the limited sample size. Since we wanted to know to which group students and facilitator belonged, differentiating between gender would result in identifying the facilitator and often the student, since the number of male participants was limited and spread over the groups. Therefore, we removed our speculations about gender in both the method and discussion, and we limited our explanation for not capturing gender to the limited sample size.
- RESULTS: The feedback of the reviewer has showed us that in making our results section more concise to address the word limit, several nuances in our explanations were lost:
  - Data on “personalization” was left out because there was no difference between groups. Yet this is indeed valuable information for the readers. We therefore updated the picture and text.
  - We decided to include the visualization for “actionable intentions”. While the other questions seemed to be misunderstood by students, the question on actionable intentions was understood but was less often answered than the other questions; we assume this is because the question was placed at the end of the survey. We decided enough data was present for this pilot study to provide readers with our insights. We furthermore elaborated on this in the explanation of the RQ3 Results.
  - The reason for not visualizing the results for all subcodes on the retrospection system in Figure 3 was left out. While the requirements for the reflection format and collection medium came back in the qualitative data, the survey data show that the requirements set up for the retrospection system were less clear and important for students. The question about the degree to which the retrospective tool makes it possible for the student to trace or see their individual journey without limitations, judgments, or prescriptive visions of how it should be (i.e. individuality) was removed from the analysis as several students gave the answer “I don’t understand this question”. Furthermore, the questions addressing the degree to which the tool allows students to see their own development over time without comparing to others (i.e. comparing with oneself) and the degree in which students can take a broader, helicopter view about their own development over time (i.e. meta level reflection) were answered similarly. Therefore, these two questions were combined into a new item titled personal development (created by taking the average of each student’s score for meta level reflection and comparing with oneself). We now condensed this explanation and added it to the document, since we agree with the reviewer that this is unclear.
  - Combining the data of the subcodes into one paragraph of text for each research question, caused several problems in attributing the right results to the right group. We have attempted to resolve these problems.
  - In several cases, condensing the findings resulted in a reduction of contextualizing phrasing such as “students considered …” instead of “most/a small number/more than half/etc. students considered”. Besides the comments of the reviewer, we made sure the carefully read the article to address this in the whole paper.
- RESULTS-figure 3: The reviewer also provided suggestions to make Figure 3 approachable for readers who have not read the article or extended data. In line with the reviewer’s suggestions, we added the statements that students were asked to score with Likert scales, and we added an explanation about the anchor point of the Likert scale in the figure description.
- RESULTS-reflection analysis: The comment of the reviewer showed us that from our description it was not clear that these numbers referred to the REFLECT scores and not the Likert scales of the students. We adapted the text to accommodate for this misunderstanding.
- RESULTS/DISCUSSION: The comments of the reviewer show us that the degree in freedom of choice was not clear from our descriptions. We therefore made sure to clearly distinguish between parts of the intervention students were assigned to (either group 1, 2 or 3), and parts students could choose (the reflection format (image/text/audio/video for the students who received Intervention 1 or visual/text for the other students) and topic (e.g. which insights and how elaborately the students captured these)).
- RESULTS/DISCUSSION: We thank with the reviewer for noticing that our attempt to be clear and concise resulted in an overstatement of the findings in some instances. We adapted both the results and discussion for it to better reflect the nuanced findings. We addressed the reviewer’s comments on the balance between positive and negative quotes extracted from our data;  the value of the reflection format; the actionable intentions of students; the benefits of the reflection materials to outbalance the time and energy; our suggestion for using a combination notes to self and scoring in future research; and our previous claim that the study ‘confirms’ instead of ‘suggests’ that there will always be students who do not see value in reflection.
- DISCUSSION: We appreciate questions such as ‘What percent or relative proportion of comments were positive vs negative?’. We do agree such questions are interesting, but such quantifications don’t align with our chosen method of thematic analysis. Quantification such as the one suggested in this comment are more aligned with a content analysis, which is commonly used with large, unorganized datasets. The other question presented by the reviewer ‘Were there characteristics that predicted which students valued reflection vs which did not?” cannot be answered with our data since we did not capture student characteristics. This is therefore an aspect we address in the Strengths and limitations section.
- DISCUSSION: We added the useful suggestions for further research provides by the reviewer, including more research with a study design that allows students to mix and match reflection-collection-retrospection methods.
- DISCUSSION: We addressed the comment of the reviewer that we cannot draw conclusions on sex and gender since we did not measure those. These conclusions were indeed provided as inspiration and not meant as conclusion. We therefore changed our description to a better formulate it as a suggestion for future research.

# Reviewer report: Reviewer response for version 1

**DOI:** 10.21956/mep.22445.r44283

**Published:** 2025-11-26

**Authors:** Prof. Irma Manjavidze M.D.,PhD., Associate Professor. Dali Chitaishvili M.D., PhD.

**Affiliations:** 1 Head of Department of Clinical Skills and Multidisciplinary Simulation, Tbilisi State Medical University, Tbilisi, Georgia; 2 Head of Clinical Skills and Multidisciplinary Simulation Department, Tbilisi State Medical University, Tbilisi, Georgia

This is a well-designed mixed-methods study that provides valuable insights into how medical students experience different formats for reflection, retrospection, and portfolio work related to Professional and Personal Development (PPD). The study benefits from co-design with students, strong participation rates, and clear visual diagrams that make the intervention easy to follow. The integration of qualitative and quantitative findings is thoughtful and adds depth to the results. Overall, the paper is timely and relevant to discussions about meaningful reflection and professional identity formation in medical education.

**1. Introduction**

- **Rationale for mixed-methods:** Clearly state why a convergent mixed-methods design was chosen and how combining qualitative and quantitative approaches enhances understanding of PPD reflection practices.

**2. Methods**

- **Facilitator influence:** Include a short reflection on how using senior students or recent graduates as facilitators might have influenced openness, depth of reflection, or responses (e.g., potential social desirability bias).
- **REFLECT rubric adaptation:** Add a brief paragraph explaining why the REFLECT rubric was modified for audio and visual reflections and discuss implications for validity and comparability with other studies.
- **Thematic analysis clarity:** Streamline the analytic workflow: describe how deductive and inductive codes were merged, how disagreements were resolved, and how final subcodes were derived.
- **Workload/feasibility:** Consider adding approximate time demands for workshops, home assignments, and retrospection activities. This will help educators assess the feasibility of implementation in other contexts.

**3. Results**

- **ANOVA interpretation:** Reword the description of the p = 0.057 result. Rather than calling it “almost significant,” describe it as non-significant and emphasize descriptive trends or practical meaning.
- **Digital platform results:** Distinguish between limitations specific to Miro versus limitations common to digital reflection platforms in general.

**4. Discussion**

- **Transferability and contextual factors:** Add a brief reflection on how institutional culture, coaching practices, or curriculum structure might influence applicability elsewhere.
- **Mixed-methods integration:** Include 1–2 sentences explaining how joint displays and narrative weaving enriched the interpretation of findings.
- **Longitudinal implications:** Discuss how compressing the intended six-month retrospection period may have influenced student experiences and consider suggestions for future research on timing effects.
- **Platform design implications:** Based on student feedback in joint displays, specify essential functional requirements for future digital tools (e.g., multimedia support, overview structure, ease-of-use).

**5. Structure & Style**

- **Condense overly detailed sections:** Some parts of the Methods and Discussion are very detailed or overlapping. Tightening these sections will improve readability while keeping transparency.
- **Typographical and grammatical corrections:** There are a few minor typographical errors  For example:
  – In the first paragraph of Method, third sentence, “epmployed” should be “employed.”
  – In the last paragraph of Method, fourth sentence, “The second and thirs author…” should be corrected to “The second and third author….”
  – In the second paragraph of the *Discussion*, first sentence, change “me meaningful” to “be meaningful.”

**Verdict:** Approve with Reservations.

 This is a robust and relevant study that makes a significant contribution to medical education. The main revisions involve clarifying methodological points, refining the interpretation of borderline statistical results, and tightening some sections for readability. Including moderate points like workload, longitudinal considerations, and platform design will make the report more constructive for the authors.

Have any limitations of the research been acknowledged?

Partly

Is the work clearly and accurately presented and does it cite the current literature?

Partly

If applicable, is the statistical analysis and its interpretation appropriate?

Partly

Are all the source data underlying the results available to ensure full reproducibility?

Partly

Is the study design appropriate and is the work technically sound?

Yes

Are the conclusions drawn adequately supported by the results?

Yes

Are sufficient details of methods and analysis provided to allow replication by others?

Partly

Reviewer Expertise:

Medical Education, Simulation based learning, Pediatrics, Obstetrics Gynecology

We confirm that we have read this submission and believe that we have an appropriate level of expertise to confirm that it is of an acceptable scientific standard, however we have significant reservations, as outlined above.

## References

[1] : Capturing Reflections for Personal and Professional Development in Medical Education: A Mixed Methods Study. *MedEdPublish* .2025;15: 10.12688/mep.20957.1 10.12688/mep.20957.1 41909087 PMC13019040

# Response

**Published:** 2026-02-16

**Authors:** Deanne Spek

**Affiliations:** Psychiatry, Universitair Medisch Centrum Utrecht, Utrecht, Utrecht, The Netherlands

We thank the reviewers for their clear and useful feedback. Below, we outline how we addressed each of their comments. **1. Introduction** We explained the rationale for the mixed methods design at the end of the introduction. Building on the suggestion of the reviewers, we also included an explanation for why we put more emphasis on the QN data.

**2. Methods** **Facilitator influence:** To prevent overlap, we incorporated this useful suggestion of the reviewers in the discussion section. **REFLECT rubric adaptation:**  We provided more explanation of why and how the RUBRIC was modified. The implications of the use of this rubric were addressed in the strengths and limitations section of the discussion. To prevent overlap, we therefore elaborated on the limitation of adapting the RUBRIC in the discussion section. **Thematic analysis clarity: **The reviewers asked to describe how deductive and inductive codes were merged, how disagreements were resolved, and how final subcodes were derived. We already explained our inductive and deductive coding process in the article and how disagreements were resolved in the article. However, we added information on the subcoding process in the last two sentences of the first paragraph on data analysis. **Workload/feasibility:** We added the time demands for students in Figure 2, to provide educators with this useful information for implementation.

**3. Results** **ANOVA interpretation:** We changed our framing of the non-significant result and explained we did perform a Tukey post-hoc test because our limited sample size may have influenced the “almost significant” result. **Digital platform results: **The comment of the reviewers showed us that our current attempt to distinguish between Miro-specific and general applicable feedback was not clear. We therefore changed our description in the last few sentences of the collection medium.

**4. Discussion** **Transferability and contextual factors:** We added a brief comment on the transferability of our findings in the strengths and limitations section of the discussion. We think further decisions about generalizability will have to be made by the reader, who can compare their setting to our description in the section on setting and participants. **Mixed-methods integration:** We added details on the specific usability of the joint displays and narrative weaving in the strengths and limitations section of the discussion. **Longitudinal implications:** We added this suggestion of the reviewers to the strengths and limitations section where we already addressed the effect of providing this education in a condensed format on student experiences of the usefulness of the education. **Platform design implications:** We thank the reviewers for this suggestion. However, we think further elaboration on the online platform does not match scope of this article. Further recommendations could be extracted from our data for all the aspects of the interventions (the reflection format, collection medium and retrospection system). Our sample size makes further generalization difficult; further studies will be needed to learn more about detailed aspects of the interventions.

**5. Structure & Style** We reviewed the methods and discussion sections to make them more concise and remove the overlap. In the methods section, we removed detailed descriptions of our approach and deleted repetition regarding the design principles. In de discussion section we removed repetition of the implications of the online platform used and condensed sections which had the same conclusion (i.e., more freedom for reflection). We appreciate that the reviewers point out several spelling/grammar mistakes and we have carefully reread the whole article to remove any additional mistakes.
